# Microcin H47 System: An *Escherichia coli* Small Genomic Island with Novel Features

**DOI:** 10.1371/journal.pone.0026179

**Published:** 2011-10-11

**Authors:** María F. Azpiroz, Thais Bascuas, Magela Laviña

**Affiliations:** Sección Fisiología y Genética Bacterianas, Facultad de Ciencias, Montevideo, Uruguay; Baylor College of Medicine, United States of America

## Abstract

Genomic islands are DNA regions containing variable genetic information related to secondary metabolism. Frequently, they have the ability to excise from and integrate into replicons through site-specific recombination. Thus, they are usually flanked by short direct repeats that act as attachment sites, and contain genes for an integrase and an excisionase which carry out the genetic exchange. These mobility events would be at the basis of the horizontal transfer of genomic islands among bacteria.

Microcin H47 is a ribosomally-synthesized antibacterial peptide that belongs to the group of chromosome-encoded microcins. The 13 kb-genetic system responsible for its production resides in the chromosome of the *Escherichia coli* H47 strain and is flanked by extensive and imperfect direct repeats. In this work, both excision and integration of the microcin H47 system were experimentally detected. The analyses were mainly performed in *E. coli* K12 cells carrying the microcin system cloned in a multicopy plasmid. As expected of a site-specific recombination event, the genetic exchange also occurred in a context deficient for homologous recombination. The DNA sequence of the attachment sites resulting from excision were hybrid between the sequences of the direct repeats. Unexpectedly, different hybrid attachment sites appeared which resulted from recombination in four segments of identity between the direct repeats. Genes encoding the trans-acting proteins responsible for the site-specific recombination were shown to be absent in the microcin H47 system. Therefore, they should be provided by the remaining genetic context, not only in the H47 strain but also in *E. coli* K12 cells, where both excision and integration occurred. Moreover, a survey of the attachment sites in data banks revealed that they are widely spread among *E. coli* strains. It is concluded that the microcin system is a small island –H47 genomic island- that would employ a parasitic strategy for its mobility.

## Introduction

The increasing sequencing of complete bacterial genomes revealed the existence of a flexible genetic content that varies even among closely related strains. In the chromosome, this variable genetic information is gathered in defined regions of up to 200 kb called genomic islands (GI). Their coding capacity is very diverse, including traits related to virulence, symbiosis, antibiotic resistance, complex nutrient metabolism, among other functions of the secondary metabolism [1]. Many GIs have been shown to insert into and excise from the chromosome through site-specific recombination events that underlie the island gain, loss and transfer [2], [3].

The mobility of GIs reproduces the site-specific recombination events characteristic of temperate phages following the lisogenic pathway. It requires the presence of attachment sites and the participation of specific recombination functions carried out by an integrase and an excisionase. Thus, most GI are flanked by short direct repeats that act as attachment sites left and right (*attL* and *attR*) and contain the genes for the corresponding integrase and excisionase [4]–[7]. The mobility of a GI implies several sequential events that may include its horizontal transfer. First, the island integrated into the chromosome excises through site-specific recombination between its flanking repeats, a process operated by the corresponding integrase-excisionase pair. This occurs at a very low frequency and results in the chromosome without the island and the island as a circular non-replicative intermediate. Each molecule contains a hybrid sequence resulting from the recombination of the *attL* and *attR* sites: the attachment in the chromosome (*attC*) and the attachment in the excised island (*attI*). Then, the GI as an independent molecule may be horizontally transferred to a recipient bacterial cell. Transduction and conjugation have been described to mediate the transfer of some GIs [8]–[11]. Once in the recipient cell, the GI integrates into the chromosome through site-specific recombination between its *attI* and a resident *attC*, a process carried out by the island-encoded integrase. Thus, the original condition of the GI is reproduced, but now in the context of a new host.

The genetic system responsible for the production of the antibacterial peptide microcin H47 (MccH47) in the *Escherichia coli* H47 strain resides in the chromosome and is flanked by a direct repeat [12]. This structure suggested that the MccH47 genetic system could be a genomic island able to be mobilized by site-specific recombination.

**Figure 1:**
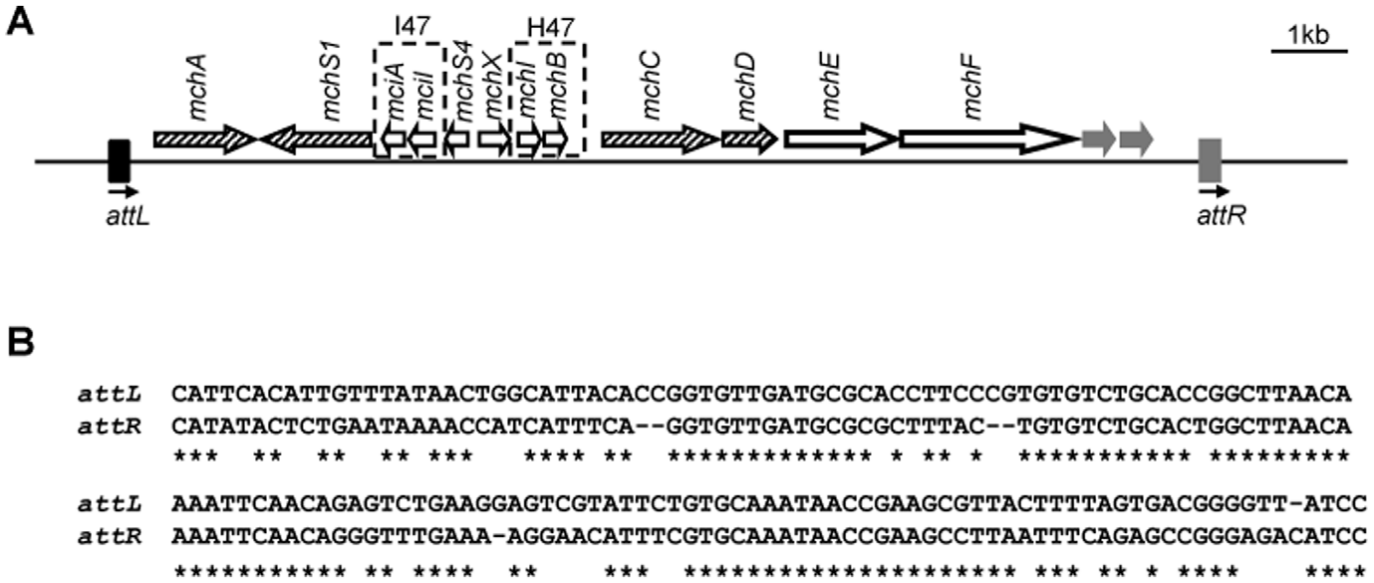
Microcin H47 genetic system, direct repeats and adjacent regions. A. Representation of the 16,823 bp DNA segment from the *E. coli* H47 chromosome (accession number: AJ009631) that was cloned into the recombinant plasmids pEX100, pEX2000 and pEX4. Pairs of immunity-activity genes for the encoded microcins are boxed. Truncated genes related to microcin M are shown as grey arrows. Hatched, maturation genes; bold, secretion genes; white outside the boxes, regulatory genes. The imperfect direct repeats flanking the MccH47 system are shown as black (*attL*) and grey (*attR*) rectangles. B. Alignment of the direct repeats flanking the MccH47 genetic system. The alignment was performed with the program Lalign [22]. Asterisks indicate nucleotide identities.

MccH47 is a ribosomally-synthesized antibacterial peptide that belongs to the group of chromosome-encoded microcins [12]. The MccH47 genetic system contains all genes necessary for the peptide production, for its post-translational maturation and secretion and for an immunity peptide that protects the producing cell against the MccH47 action. It also contains genes for a second activity, microcin I47, whose production is only detected in iron deprivation conditions. A chromosomal 16,823 bp DNA segment containing the MccH47 system plus adjacent regions at both sides has been cloned in several plasmid vectors (Fig. 1A) [12]. The nucleotide sequence of this segment revealed the presence of an extensive direct repeat flanking the MccH47 system. The repeated sequences share 76% of identity and are particularly extensive, being of 148 bp (left) and 144 bp (right) [12]. They will be referred to as *attL* and *attR* (Fig. 1B).

In this work, the MccH47 genetic system is shown to be a small GI able to be mobilized by site-specific recombination in both directions of excision and integration. Surprisingly, it does not contain the genes for the recombination functions and, when introduced in a laboratory *E. coli* K12 strain, it is still mobilized. Presumably, the host context provides the required recombination trans-acting functions.

## Results

### Comparative analysis of the DNA segment containing the MccH47 genetic system

**Figure 2:**
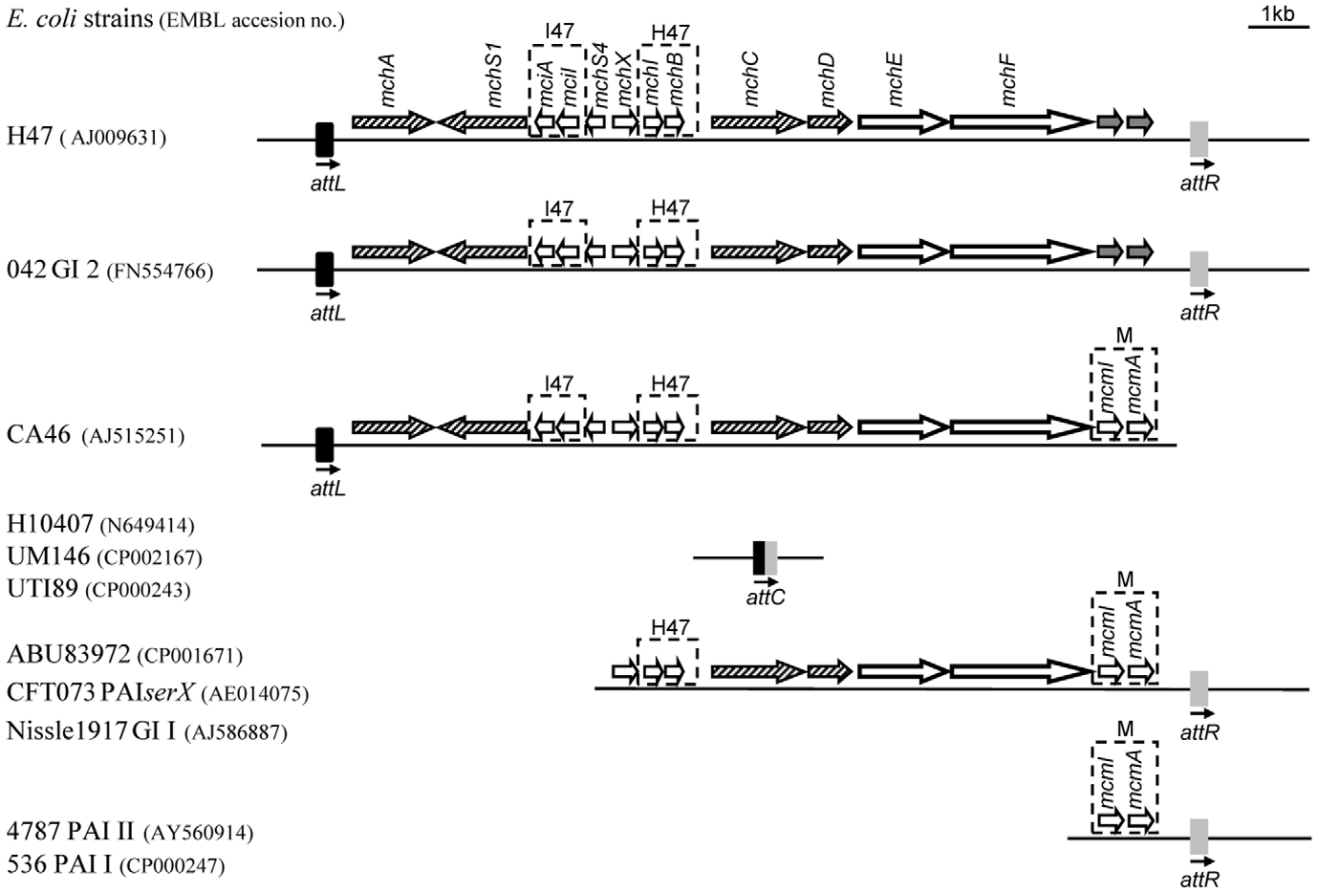
Comparison of the DNA segment containing the MccH47 genetic system. The 16,823 bp DNA segment containing the MccH47 system is represented above. Examples of DNA segments exhibiting high levels of identity (>85%) with the reference sequence are shown below. Genes from the MccH47 system and their homologues in other organisms are shown with arrows as in Figure 1. Wild-type immunity-activity genes for the encoded microcins are boxed. PAI, pathogenicity-associated island.

Comparisons with sequences in data bank were performed with the BLASTN program [13]. First, the sequence of each direct repeat flanking the MccH47 system was analyzed. High levels of identity were found (>75%) with chromosomal sequences from enterobacterial strains, most of them located into genomic islands of pathogenic *E. coli* strains. As expected, the same sequences matched both repeats, although with different scores. Some of them were more related to the left direct repeat (*attL*), others to the right one (*attR*), and still others appeared to be hybrid between both. These hybrid sequences were categorized as empty chromosomal attachment sites (*attC*). Then, the analysis focused on the 16,823 bp DNA segment containing the MccH47 genetic system. Three main types of chromosomal genetic structures were identified: the MccH47 system integrated between *attL* and *attR*; different extensions of the MccH47 system accompanied by the *attR* but lacking the *attL*, and the putative *attC* site. Some examples are shown in Figure 2. The entire query sequence was found almost identical into the GI 2 of the enteroaggregative *E. coli* strain 042 [14]. It could also be present in the *E. coli* strain CA46, which contains the MccH47 system limited by an *attL*-like sequence. However, the presence of an *attR* remains unknown because the release does not extend downstream of *mcmA*. Partial MccH47 sequences keeping the *attR* site were mainly found in extraintestinal pathogenic *E. coli* strains such as CFT073. Finally, the *attC* site was identified mostly in intestinal and extraintestinal pathogenic *E. coli* strains, and in the *Salmonella enterica* Heidelberg SL476 strain. In principle, all the homologies tended to extend to the adjacent sequences outside the repeats.

### Excision of the MccH47 genetic system

**Figure 3:**
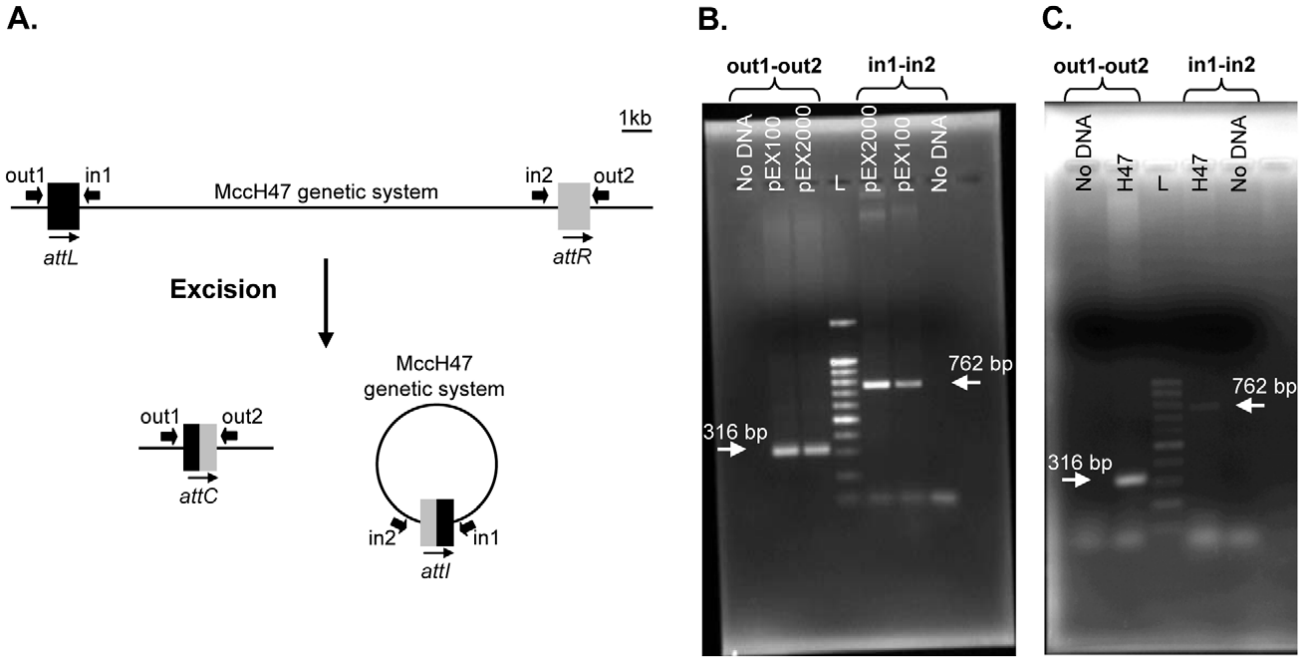
Excision of the MccH47 genetic system. A. Schematic representation of the PCR approach performed to detect the excision of the MccH47 system. Amplification products containing the *attC* and *attI* were obtained with primer pairs out1-out2 and in1-in2, respectively. B. Detection of the MccH47 system excision from plasmids pEX2000 and pEX100 with the indicated primer pairs in the context of RYC1000 cells. L: 1,5 kb DNA ladder (Bioron). C. Detection of the MccH47 system excision from the *E. coli* H47 chromosome. L: 100 bp DNA ladder (Bioron).

The excision of a GI from the chromosome is an event that usually occurs at a very low frequency. Therefore, to detect the possible excision of the MccH47 genetic system a PCR approach was employed. A set of primers was designed to hybridize at each side of the *attL* and *attR* sites. PCR reactions were performed combining the primers so that amplicons would be produced only if excision had occurred (Fig. 3A). The excision was assessed in the *E. coli* H47 strain and in *E. coli* K12 strains (BZB1011 and RYC1000) carrying the MccH47 system cloned in different multicopy plasmid vectors (pEX100, pEX4 or pEX2000). Considering that the study of the mobility of GIs in recombinant contexts is a novel experimental approach, the three plasmids mentioned above were employed in order to discard possible peculiarities of the plasmid vector context. For its part, RYC1000 provided a *recA* deficient context, a condition that was important to rule out the possible involvement of homologous recombination. The result was that PCR products of the expected size were obtained in all the genetic contexts assayed (Fig. 3B and data not shown). In the case of the H47 strain, a nested PCR was required to detect excision (Fig. 3C). Therefore, the MccH47 system excised from the chromosome -or from the recombinant plasmids- as would be expected of a GI submitted to a site-specific recombination between its flanking direct repeats, the *attL* and *attR* sites.

### The recombination site

**Figure 4:**
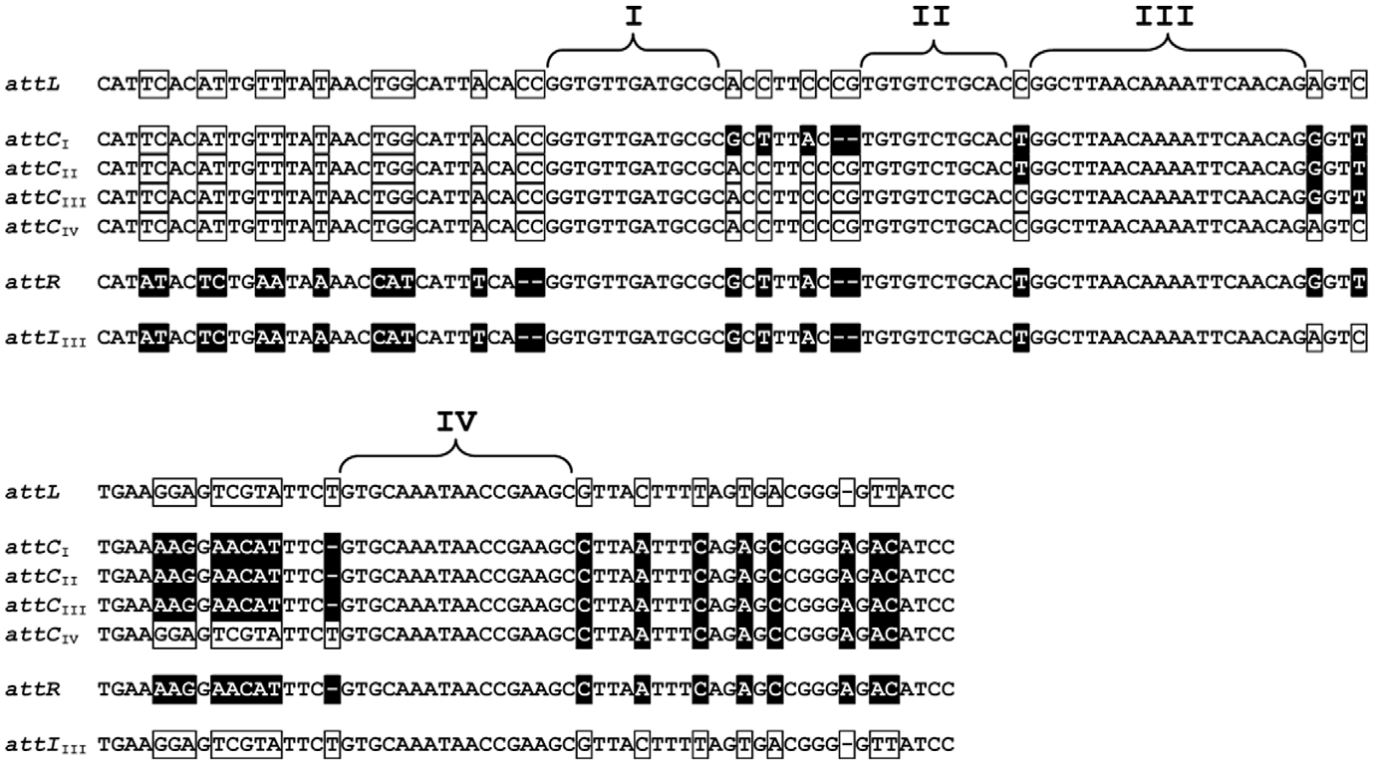
Alignment of *attL* and *attR* with the different *attC* and *attI* sites obtained. Specific nucleotides of *attL* are in clear boxes and those of *attR* in black boxes. The attachment sites resulting from recombination in different regions (I–IV) are named as *attC* or *attI* followed by the roman number corresponding to the site of recombination.

The direct repeats flanking the MccH47 system are extensive and imperfect. They contain four stretches of total identity separated by nucleotide differences. These stretches were named I to IV and, supposedly, the site of recombination would be located in one of them (Fig. 4). The PCR products containing the *attC* and the *attI* sites were separately cloned in a plasmid vector to construct pOUT and pIN, respectively, as explained in Materials and Methods. The nucleotide sequence of pOUT revealed a hybrid sequence between the direct repeats resulting from a crossing in the region of identity II (Fig. 4). The insert in plasmid pIN was also hybrid but, unexpectedly, it exhibited a different site of recombination, located in region III (Fig. 4). In view of this difference, a total of 20 pOUT plasmids independently obtained were sequenced. Four different *attC* sites were thus found, resulting from the recombination in the four extended identical regions I-IV (Fig. 4). Since the cloned PCR products could be different in their sequence, the designation of plasmids pOUT and pIN included the roman number corresponding to the region where recombination had occurred, e.g. pOUT_II_. The four types of pOUT appeared with the following frequencies: one pOUT_I_, seven pOUT_II_, ten pOUT_III_ and two pOUT_IV_.

These results indicated that the PCR products obtained in the excision assays were heterogeneous. Therefore, if they were directly sequenced, a series of overlapping sequences would appear at the sites of nucleotide differences depending on the site of recombination. This was determined in a PCR product obtained using primers out1 and out2, i.e. in a mix of *attC* sequences. Prior to sequencing, the PCR product was run in an agarose gel and the corresponding band was purified in order to eliminate the template that had been employed in the reaction. DNA sequencing was performed using primer out1. At the beginning, the reading was unique, but past each region of recombination, an additional reading appeared in some positions, exactly in those where two or more nucleotides would be expected if the DNA were a mix of sequences that had recombined in different sites. Thus, the four regions of recombination I–IV were recognized (data not shown). In agreement with the results presented above, the main reading of the sequence changed from *attL* to *attR* past the recombination site III, indicating that most of the excision events had taken place at this site.

Therefore, the nature of the direct repeats as attachment sites for site-specific recombination was confirmed. Moreover, it was found that they are complex and contain multiple recombination sites.

### Integration of the MccH47 system

**Figure 5:**
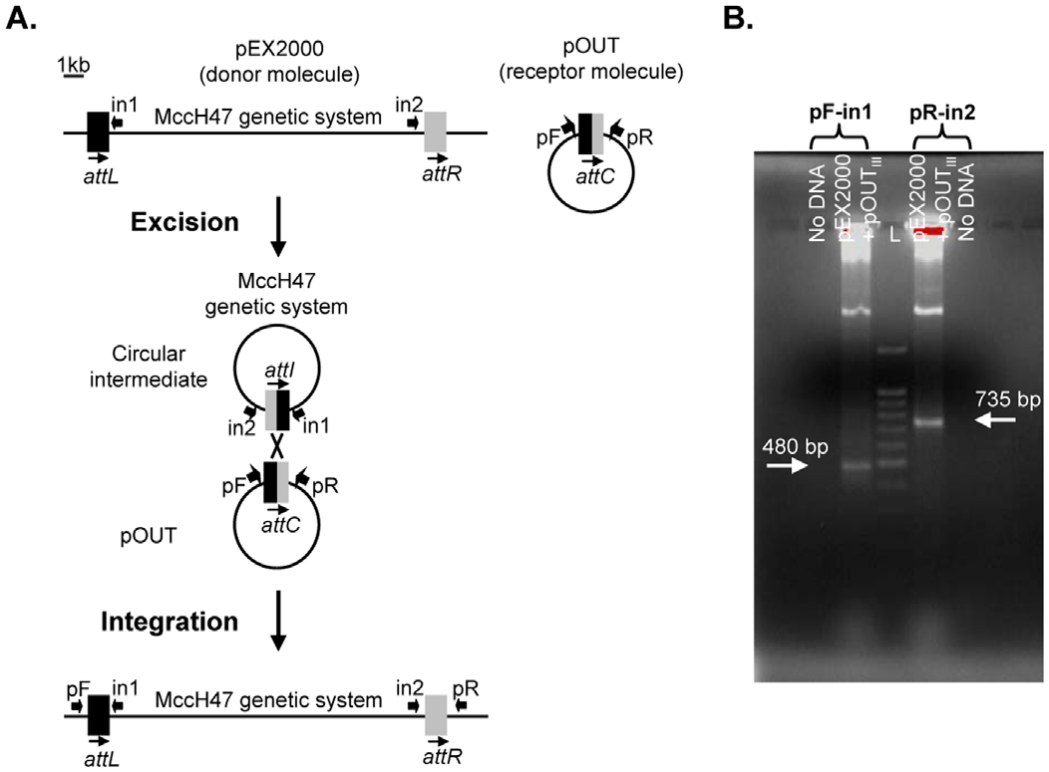
Integration of the MccH47 genetic system. A. Schematic representation of the PCR approach performed to detect the integration of the MccH47 system. Amplification products containing the *attL* and *attR* sites flanking the MccH47 system integrated into pOUT plasmid were obtained with primer pairs pF-in1 and pR-in2, respectively. pR, M13 reverse primer; pF, M13 forward primer. B. Detection of the MccH47 system integration into pOUT_III_ plasmid with the indicated primer pairs. L: 1,5 kb DNA ladder (Bioron).

A PCR approach was also employed following the design depicted in Fig. 5. The donor molecule was plasmid pEX2000 and two alternative recipient molecules were employed, pOUT_II_ and pOUT_III_, carrying two versions of *attC*. Thus, the integration was assayed in two contexts, RYC1000(pEX2000, pOUT_II_) and RYC1000(pEX2000, pOUT_III_), being both *recA* deficient. Primers in1 and in2 were combined with primers annealing with the plasmid vector (forward and reverse) so that amplicons would only be produced if the MccH47 system had integrated into the *attC* of the pOUT plasmid (Fig. 5A). In both contexts assayed, PCR products of the expected size were obtained (Fig. 5B and data not shown), thus confirming the ability of the MccH47 system to integrate into the two versions of the *attC* site.

### Recombination functions responsible for the mobility of the MccH47 system

**Figure 6:**
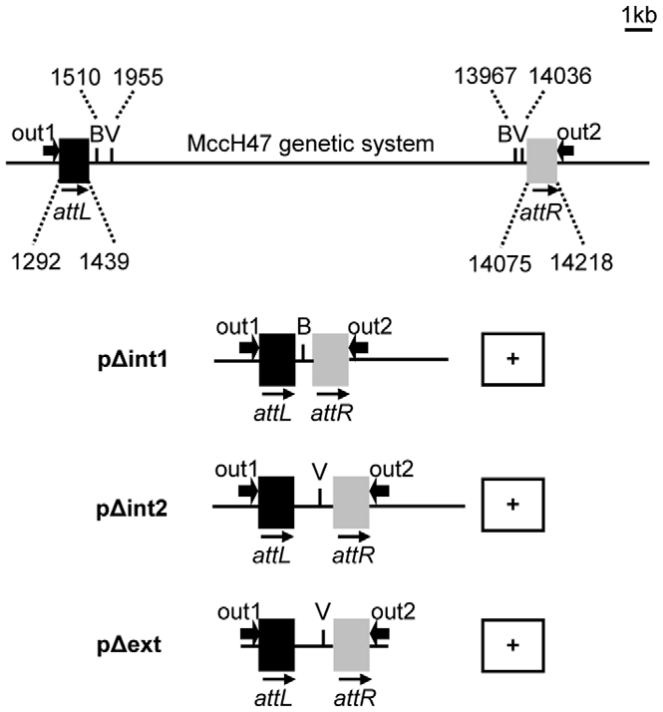
Genetic constructions derived from plasmids carrying the MccH47 system. Constructions are derived from plasmids pEX4 and pEX100, both carrying the 16,823 bp DNA segment shown above. Only the most external restriction sites for the enzymes BspHI (B) and EcoRV (V) are shown. Nucleotide numbers are indicated for the start and end of *attL* and *attR*, and for the restriction sites. Plasmid pΔext keeps 71 bp and 95 bp DNA segments from the left and right adjacent regions, respectively. “+”, positive PCR reactions with primers out1 and out2, indicating the occurrence of excision.

At this point, it was evident that the MccH47 system was excised and integrated through site-specific recombination. Therefore, trans-acting activities should operate the genetic exchange, namely a site-specific recombinase and an ancillary excisionase. GIs usually contain their specific mobility genes; however, none of the genes of the microcin system could in principle be related to such function. Therefore, deletion derivatives of plasmids pEX100 and pEX4, pΔint1 and pΔint2, respectively, were constructed (Fig. 6). In both cases, almost all the MccH47 genetic information was eliminated (pΔint1 was more exhaustive to the left and pΔint2 to the right, as represented in Fig 6) while the direct repeats and adjacent regions flanking the antibiotic system were retained. Then, excision assays were performed with these constructions carried by RYC1000 cells. Only the PCR reaction with primers out1 and out2 was carried out since the plasmids lacked the sequences complementary to primers in1 and in2. The appearance of the expected 316 bp amplicon indicated that excision still occurred in both cases. Therefore, the genetic determinants responsible for the MccH47 system excision should be located outside the system.

Considering that excision was taking place in the *E. coli* K12 recombinant context, it seemed most likely that the recombination genes would be located in the external adjacent regions included in the recombinant plasmids. To assay this possibility, plasmid pΔext, which is a derivative of pΔint2 lacking the adjacent regions of the MccH47 system at both sides, was constructed (Fig. 6). Excision was now assayed in RYC1000 cells carrying this plasmid and, surprisingly, the 316 bp amplicon appeared again. These results clearly indicated that genes for the MccH47 system mobility should be located in a chromosomal region unlinked to the system. Moreover, since recombination occurred in *E. coli* K12 cells, it should be deduced that this foreign context was able to provide the required trans-acting functions.

## Discussion

Results presented in this work indicate that the MccH47 system is a small 12,635 bp GI able to be mobilized by site-specific recombination. The name of H47 GI is now proposed for this genetic structure.

Both events of excision and integration of the H47 GI were experimentally determined. Of note, although excision was confirmed in the original *E. coli* H47 strain, the great majority of the experiments was performed in the context of laboratory *E. coli* K12 strains. Indeed, an *E. coli* H47 chromosomal region containing the island and adjacent sequences had been previously cloned in several multicopy plasmid vectors. These genetic constructions, propagated in an *E. coli* K12 strain, greatly facilitated the study of the H47 GI mobility. A clear advantage was the easier detection of the island excision, most probably due to the high copy-number of the molecules containing it. In fact, excision was detected after a single amplification in the recombinant context while a nested PCR reaction was needed in the case of *E. coli* H47. As to the integration of the H47 GI, it was only assayed in the recombinant context and resulted more difficult to detect than excision. This could be due to the fact that experiments implied both the H47 GI excision from a donor molecule and then its integration into a recipient molecule, i.e. the sequential occurrence of two infrequent events. In addition, all these genetic exchanges could be observed in an *E. coli* K12 strain deficient for homologous recombination, a result that directly indicated that the genetic exchange was being operated by site-specific recombination.

The excision of the H47 island was clearly detected and the attachment sites of the resulting products, *attC* and *attI*, were cloned. Thanks to the nucleotide differences between the attachment sites flanking the island, *attL* and *attR*, the recombinant sequences could be recognized in the excision products. The DNA sequence of several *attC* sites independently cloned revealed that recombination could occur in four different regions of the direct repeats. This complexity would be at the basis of the unusual extension of the attachment sites of the H47 GI. In this sense, an island has been recently described in *Salmonella enterica* serovar enteritidis (defective prophage-like element φSE14) that is also flanked by long imperfect repeats which contain different regions of recombination [15].

Considering that site-specific mobilization of genomic islands depends on the action of a specific integrase and of an ancillary excisionase, genes coding for these trans-acting elements were searched for. GIs usually carry these genetic determinants; however, we could demonstrate that the H47 GI lacks such information. Moreover, the adjacent chromosomal regions cloned together with the island in the recombinant plasmids used did not contain the required genetic information either. These results were highly unexpected and most informative because they signified that the trans-acting elements operating the recombination were being provided by the *E. coli* K12 context. Moreover, given the existence of multiple recombination sites, it could be speculated that more than one integrase-excisionase pair could perform the genetic exchange. In any case, the H47 GI could be seen as a genetic element employing a parasitic strategy for its mobility.

Comparative sequence analyses against data banks revealed that some *E. coli* strains contain in their chromosome the H47 GI integration site *attC*, indicating that this island would have the potential to integrate into the chromosome of these strains. In this sense, the H47 GI was found integrated into the genomic island 2 of *E. coli* 042, an enteroaggregative strain whose genome was recently sequenced [14]. It is interesting to note that most of the *attC* sites found in data banks are of the *attC*_III_ type and only one is *attC*_II_, i.e. those types most frequently produced after excision of the H47 GI in the *E. coli* K12 context. This strongly suggests that the recombinases involved in the site-specific recombination events would be broadly distributed in the *E. coli* species.

In conclusion, the MccH47 genetic system has proven to be a small GI able to be mobilized by site-specific recombination. As such, it exhibits novel features related to its structure and mobility functions. It is flanked by particularly extensive direct repeats which are composed of a tandem of four recombination sites. In addition, the H47 GI does not contain the determinants for the required trans-acting recombination proteins. These should be provided by the host chromosome where the H47 island integrates, as was shown in an *E. coli* K12 recombinant context. In sum, the H47 genomic island displays an original parasitic strategy for its mobility.

## Materials and Methods

### Bacterial strains, plasmids, and growth conditions

*E. coli* H47 is a natural producer of MccH47 [16]. *E. coli* K12 strains from our laboratory collection were used: RYC1000 (*araD gyrA ΔlacU169 rbs recA relA rpsL thiA*), DH5α (*endA gyrA hsdR ΔlacU169 recA relA supE thiA*) and BZB1011, which is a *gyrA* derivative of W3110, a strain whose genome has been completely sequenced [17]. Recombinant plasmids carrying the MccH47 system included in a 16,823 bp chromosomal DNA segment from strain H47 were: pEX100 (pACYC184-derivative), pEX2000 (pBR322-derivative) and pEX4 (pUC13-derivative) [12], [18]. Plasmid pUCYC5, a medium copy-number vector carrying the polylinker-*lacŹ* region from pUC13, was used as the cloning vector throughout the study [19]. Bacteria were grown in Luria-Bertani rich medium [20]. Antibiotics were added to media at the following final concentrations: ampicillin, 100 µg/ml; chloramphenicol, 60 µg/ml. The chromogenic indicator of β-galactosidase activity X-gal (5-bromo-4-chloro-3-indoyl-β-D-galactopyranoside) was added to media at the final concentration of 20 µg/ml.

### Molecular biology techniques and plasmid construction

Cloning experiments and extraction of plasmid DNA were as already described [21]. Total genomic DNA from H47 strain was extracted with the “Wizard genomic DNA purification system” (Promega). To detect the excision of the MccH47 system the following primers were used: out1 (5′-CCGTTCATTTTCCTGCTGACCC-3′) and out2 (5′-TCTGTTGCCCGTTGATGTTTCCT-3′) for the “out reactions”, and in1 (5′-GTTTGTAGGAGCTTTCTTTTTTG-3′) and in2 (5′-CGCTGATGACTGTTTTTATGTTG-3′) for the “in reactions” (Fig. 3). To detect the integration of the MccH47 system, primers in1 and in2 were appropriately combined with the M13 forward and reverse primers (Fig. 5). The M13 primers anneal to pUCYC5 sequences adjacent to the polylinker. PCR amplifications were performed using U-Taq DNA polymerase (SBS Genetech) in a total volume of 30 µl. Reaction mixes contained 1x buffer, 2 mM MgCl_2_, 200 µM of each deoxynucleotide triphosphate, 500 nM of each primer, 0.6 U of DNA polymerase and high quantities of template DNA. Depending on the type of experiment, the amount of DNA template was added as follows: to detect excision, 200 ng of plasmid DNA or 300-400 ng of genomic DNA from strain H47; to detect integration, 2–4 µg of total plasmid DNA. Standard conditions for amplification were: 2 min at 94°C, 30 cycles of incubation at 94°C for 30 sec, annealing temperature for 30 sec (58°C in “out” reactions, 53°C for the “in” reactions and 49°C to detect integration), 72°C for 30 sec, and a final extension step at 72°C for 2 min. The same conditions were employed for nested PCR amplifications, using 10 µl of the first PCR reaction as template for the second one. PCR products obtained for sequencing purposes were purified with “QIAEXII Gel extraction kit” (Qiagen). For cloning purposes, PCR reactions were performed with Vent polymerase (Biolabs), which has proof-reading activity and produces amplicons with blunt ends. The resulting PCR products were purified with “MinElute PCR purification kit” (Qiagen). Thus, amplicons obtained with primers out1 and out2, as well as with in1 and in2, were cloned into the unique HincII site of the pUCYC5 vector. The recombinant plasmids thus obtained were called pOUT and pIN. In all cases the cloned amplicons proceeded from PCR reactions using as template plasmid DNA extracted from RYC1000(pEX4) cells. Two additional constructions were performed to eliminate microcin genetic information from recombinant plasmids carrying the MccH47 system: plasmid pΔint1 resulted from a BspHI deletion of pEX100 and plasmid pΔint2 from an EcoRV deletion of pEX4. Finally, a PCR-segment obtained using primers out1 and out2, and pΔint2 as template, was cloned into the HincII site of pUCYC5. The resulting plasmid, pΔext, lacked almost all the *E. coli* H47 chromosomal sequences that were carried by pEX4 with the exception of the two direct repeats that flanked the MccH47 system.

### DNA sequencing

DNA sequencing was performed at the “Molecular Biology Unit” of the Pasteur Institute of Montevideo.

## References

[1] Juhas M, van der Meer JR, Gaillard M, Harding RM, Hood DW (2009). Genomic islands: tools of bacterial horizontal gene transfer and evolution.. FEMS Microbiol.

[2] Hacker J, Kaper JB (2000). Pathogenicity islands and the evolution of microbes.. Annu Rev Microbiol.

[3] Schmidt H, Hensel M (2004). Pathogenicity islands in bacterial pathogenesis.. Clin Microbiol Rev.

[4] Lewis JA, Hatfull GF (2001). Control of directionality in integrase-mediated recombination: examination of recombination directionality factors (RDFs) including Xis and Cox proteins.. Nucleic Acids Res.

[5] Lesic B, Bach S, Ghigo J, Dobrindt U, Hacker J (2004). Excision of the high-pathogenicity island of *Yersinia pseudotuberculosis* requires the combined actions of its cognate integrase and Hef, a new recombination directionality factor.. Mol Microbiol.

[6] Hochhut B, Wilde C, Balling G, Middendorf B, Dobrindt U (2006). Role of pathogenicity island-associated integrases in the genome plasticity of uropathogenic *Escherichia coli* strain 536.. Mol Microbiol.

[7] Murphy RA, Boyd EF (2008). Three pathogenicity island of *Vibrio cholerae* can excise from the chromosome and form circular intermediates.. J Bacteriol.

[8] Burrus V, Waldor MK (2004). Shaping bacterial genomes with integrative and conjugative elements.. Res Microbiol.

[9] Douard G, Praud K, Cloeckaert A, Doublet B (2010). The *Salmonella* genomic island 1 is specifically mobilized in *trans* by the incA/C multidrug resistance plasmid family.. PloS ONE.

[10] Novick RP, Christie GE, Penadés JR (2010). The phage-related chromosomal islands of Gram-positive bacteria.. Nature Rev.

[11] Waldor MK (2010). Mobilizable genomic islands: going mobile with oriT mimicry.. Mol Microbiol.

[12] Poey MA, Azpiroz MF, Laviña M (2006). Comparative analysis of chromosome-encoded microcins.. Antimicrob Agents and Chemother.

[13] Altschul SF, Madden WTL, Schaffer AA, Zhang J, Zhang Z (1997). Gapped BLAST and PSI-BLAST: new generation of protein database search programs.. Nucleic Acids Res.

[14] Chaudhuri RR, Sebaihia M, Hobman JL, Webber MA, Leyton DL (2010). Complete genome sequence and comparative metabolic profiling of prototypical enteroaggregative *Escherichia coli* strain 042.. PloS ONE.

[15] Santiviago CA, Blodel CJ, Quezada CP, Silva CA, Tobar PM (2010). Spontaneous excision of the *Salmonella enterica* serovar enteritidis-specific defective prophage-like element ϕSE14.. J Bacteriol.

[16] Laviña M, Gaggero C, Moreno F (1990). Microcin H47, a chromosome-encoded microcin antibiotic of *Escherichia coli*.. J Bacteriol.

[17] Hayashi K, Morooka N, Yamamoto Y, Fujita K, Isono K (2006). Highly accurate genome sequences of *Escherichia coli* K-12 strains MG1655 and W3110.. http://dx.doi.org/10.1038/msb4100049.

[18] Gaggero C, Moreno F, Laviña M (1993). Genetic analysis of microcin H47 antibiotic system.. J Bacteriol.

[19] Azpiroz MF, Poey ME, Laviña M (2009). Microcins and urovirulence in *Escherichia coli*. Microbial Pathogenesis.

[20] Miller JH (1992). A short course in bacterial genetics..

[21] Sambrook J, Fritsch EF, Maniatis T (1989). Molecular Cloning: a laboratory manual..

[22] Huang X, Miller W (1991). A time-efficient, linear-space local similarity algorithm.. Adv Appl Math.

